# 3D Printing Adjustable Stiffness External Fixator for Mechanically Stimulated Healing of Tibial Fractures

**DOI:** 10.1155/2021/8539416

**Published:** 2021-12-23

**Authors:** Hongwei Li, Dichen Li, Feng Qiao, Lei Tang, Qi Han

**Affiliations:** ^1^State Key Laboratory for Manufacturing System Engineering, School of Mechanical Engineering, Xi'an Jiaotong University, Xi'an, Shaanxi 710049, China; ^2^Honghui Hospital, Xi'an Jiaotong University, No. 555, Youyidong Rd, Xi'an, Shaanxi 710054, China

## Abstract

External fixation is a long-standing but well-established method, which has been widely used for the treatment of fractures. To obtain the maximum benefit from the mechanical stimulus, the stiffness of the external fixator should be adjusted properly throughout the treatment phase. Nevertheless, the lack of a valid dynamic adjustable fixation device impedes this possibility. Based on the stiffness adjustment tolerance of the healing callus, this paper proposes an active-dynamic stiffness adjustable external fixator design method to meet stiffness requirements at different stages of the tibial fracture healing process. A novel external fixator with an adjustable stiffness configuration was designed, and the finite element method was used to simulate the stress distribution between fixator and fracture gap. The stiffness adjustment tolerance was determined based on previous studies. According to this tolerance, the optimal block structure dismantling sequence was sought and the corresponding stiffness was calculated through topology optimization for the entire external fixator model. The appropriate amount of variable stiffness at the fracture gap was applied by dismantling the configuration of the block structure external fixator during the healing process. A novel patient-specific adjustable stiffness external fixator for mechanically stimulated tibial fracture reduction and therapy was proposed. This enables surgeons to tailor the construction of the external fixator frame to the clinical needs of each patient. The presented dismantling approach of the block structure to produce conformable stiffness provides a new clinical treatment strategy for tibial fractures.

## 1. Introduction

Fracture healing is a physiological multifactorial process affected by the injury characteristics and the mechanical environment and involves both biological and mechanical aspects. It has been well-documented in orthopedic research that the mechanical environment and the motion experienced by a fracture can significantly affect the healing quality [1–4]. Strain can be detrimental to fracture healing if it is disproportionate to the intended ossification process [5]. For more than 50 years, external fixation has been used for the treatment of fractures due to its attractive features that involve minimal invasiveness, maximum tailor ability, and extreme versatility [6]. The stiffness of the external fixator, which is directly related to the amount of motion possible at the fracture site, has a direct crucial impact on fracture healing. The major factor that determines the mechanical milieu of a healing fracture under external fixation, and thereby the mechanism of union, is the rigidity of the selected fixation device [7]. Both overly rigid or exceedingly flexible constructs can lead to impaired fracture repair and the development of nonunion [8]. Rigid fixation can hamper the healing process, while relatively flexible fixation can promote it. However, unstable fixation leads to excessive motion and development of nonunion [9–11]. For successful treatment, the fixator stiffness should be maintained at appropriate levels throughout the entire healing period [12]. A lot of research has been conducted to determine the mechanical properties of the fixator. However, a dynamic stiffness modulation construct that can be adjusted throughout the repair process for the future clinical care of patients with fractures is needed [5]. However, to the best of our knowledge, a patient-specific external fixator with adjustable stiffness designed to alter the mechanical characteristics according to stiffness tolerance of healing callus during the various stages of fracture healing has seldom been reported.

Our group has developed a Q-fixator, which has expanded the traditional usage with an automatic reduction function that provides accuracy, minimal invasion, stable fixation, and potential flexible stress adjustment to improve the healing of long bone shaft fracture [13, 14]. Based on the Q-fixator, this paper proposes the development of a 3D printed patient-specific adjustable stiffness external fixator to produce proportionate stiffness for fracture healing. To accomplish this goal, the stiffness adjustment tolerance for fixation stability of callus at different healing stages was first obtained, based on the review of published research. According to the stiffness adjustment tolerance and referring to the topology optimization and weight reduction processes, the dismantling path planning of the block structure external fixator designed before was performed. Subsequently, the stiffness of the block structure external fixator under the planning path was calculated, and a specific dynamic dismantling sequence was developed. The stiffness magnitude of the patient-specific fixator can be selectively modified by dismantling the block structure. Its design is detailed for the treatment of tibial fracture. A new clinical treatment strategy for tibial fractures has been developed.

## 2. Materials and Methods

### 2.1. Data Acquisition for Virtual Reduction and Fixation

#### 2.1.1. Combining Computer-Assisted Reduction and Fixation of the Patient-Specific Adjustable Stiffness External Fixator

**Figure 1:**
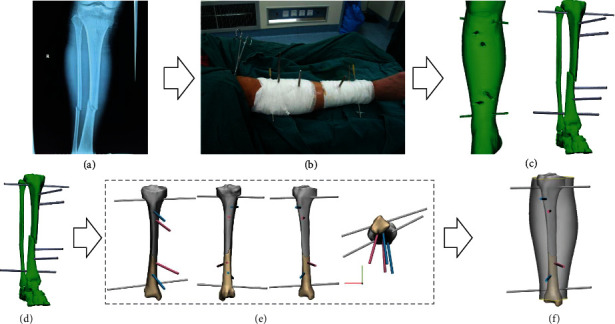
CT data acquisition and 3D modeling. (a) X-ray image of the fracture without pins; (b) patient leg after pin insertion; (c) 3D model reconstructed from CT data obtained after pin insertion; (d) virtual reduction and fixation: reconstructed 3D model of the fracture; (e) the reduction was cross-checked with reconstructed 3D fracture images and virtual reduction of the model. Two fracture blocks were reduced, and their accuracy was observed from different views; (f) final 3D geometrical model of tibial fracture with pins after reduction and fixation.

A 3D geometrical model of tibial fracture was obtained from our previous study (Figures 1). The 3D data were acquired by scanning the fracture of an adult (male, 36 years old, 69 kg, right tibial oblique fracture) at Honghui Hospital in Xi'an, China. A novel computer-assisted reduction and fixation method of the fracture was achieved using 3D printing, which has been reported in our previous papers [13, 14].

#### 2.1.2. Design of the Patient-Specific Adjustable Stiffness External Fixator

**Figure 2:**
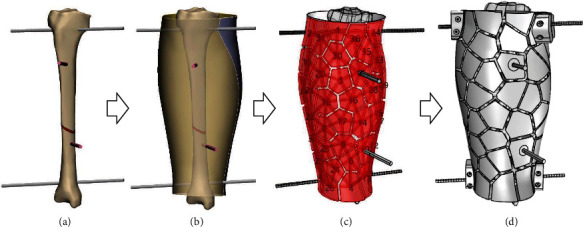
Block structure design process: (a) 3D geometrical model of tibial fracture with pins after reduction; (b) the division line of the front and back parts of the external fixation was determined based on the position of the pin passing through the fractured leg; (c) Voronoi shapes randomly distributed on the surfaces of the two parts of the fractured leg; (d) the obtained geometric model of the block structure external fixator cast.

After the virtual reduction and fixation, a 3D model in STP format of the fractured leg with pins processed by the Geomagic software was imported into Rhino to design the block structure. The division line, which divided the external fixation into front and back parts, was determined based on the position of the pin passing through the fractured leg (Figure 2(a)). Voronoi shapes were randomly distributed on the surfaces of the two parts of the fractured leg. The size of the Voronoi shapes varied between 2 and 5 cm; the thickness of Voronoi shapes is 1 cm. It is worth noting that the complete Voronoi shapes were secured at the position of the pin. After the Voronoi shapes were offset by 1 cm, their center was found, and the adjacent Voronoi center was connected by a tubular structure with a diameter of 1 cm (Figure 2(b)). A fixed structure was designed in the two sides through which the pin passed, and a geometric model of the block structure external fixator cast was obtained (Figure 2(c)).

**Figure 3:**
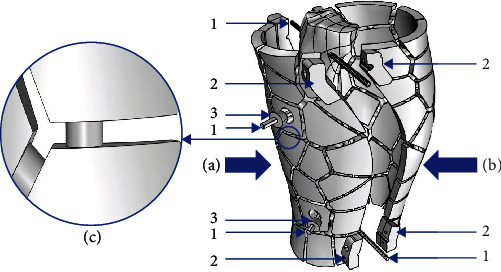
Configuration and components of the adjustable stiffness external fixator: (a) anterior frame; (b) posterior frame; (1) pin and half pin; (2) clamp with mounting holes where the mounting pins are imbedded; (3) actuators; (c) detail of the connecting rod which regulates the stiffness.

In general, the circular frame was attached to the bone through pins or partially threaded half pins. Each frame had three to four mounting holes that were used to connect the frames and bone by inserting pins through them. The two frames were connected by four parallel threaded rods. In this research, the block structure fixator frame comprises two anteroposterior main frame parts that were connected by a thread. This configuration facilitates computer-assisted reduction and fixation. The external load and fixation stability were the main issues of the mechanical environment. The construct stiffness is the pivotal factor that maintains the correct bony alignment under mechanical load. The fixator configurations can substantially change the stiffness properties of the bone-fixator system. Knowing this, an external fixator was designed such that when the stiffness of the fixator needs to be altered, this can be achieved by dismantling the connection rods attached to the block structure. As it can be seen in Figure 3, the components of the patient-specific block structure fixator model include two anteroposterior main frame parts (Figures 3(a) and 3(b)), four pins, and two actuators which can prevent the pins from loosening. The two main frame parts support the bone segments through four pins that were fixed to the central part of the Voronoi polygons and pass through the bone segment. The two parts were connected by four sets of studs and nuts placed in the clamp structure.

The pins were externally held by clamps with mounting holes and were also attached to the block structure. The stiffness was controlled by connection elements with different thicknesses (Figure 3(c)), in order to maintain the stability and stiffness of the bone structure. Since the fixation principle of this block structure fixator was similar to that of the Ilizarov apparatus, this structure can also provide stable fixation and has strong antirotation and antibending abilities, preventing the development of shear and rotational forces.

### 2.2. Validation of Stress Distribution between External Fixator and Fracture Gap during Fracture Healing

FE simulations of bone fracture healing were performed to investigate the stress distribution between external fixator and fracture gap during the healing process. An external fixator was adopted to ensure that the stress on the fracture gap can be adjusted by dismantling the block structure. It has been demonstrated that the axial load is shared by the fracture callus and the support device proportionally to the relative stiffness of the fixator and the callus [15]. However, for the novel patient-specific external fixation configuration developed in this study, the stress distribution at the fracture gap and on the external fixation is unknown. The simulation of the stress distribution and fracture healing process is valuable for subsequent clinical applications, such as the assessment of diagnosis and treatment strategies.

In the present study, the stress distribution on the fracture gap and external fixation can be validated by the following methods. A random point at the same height on the fracture gap and the external fixation cast can be taken, and the stress values at different stages of fracture healing (load step in finite element analysis) are observed and recorded. The sum of the stress values on the fracture gap and external fixation cast at different stages of fracture healing can be calculated. If the sum of stress values obtained from a random point from the fracture gap and the external fixation cast at the same height is equal to the axial load applied to the tibial plateau, it can be concluded that the axial load is shared by the fracture callus and the support device proportionally to the relative stiffness of the fixator and the callus. If they are not equal to the axial load applied to the tibial plateau or the stresses on the fracture gap and the external fixation cast do not change proportionally at different stages of fracture healing, it can be concluded the axial load is not equally shared by the fracture callus and the support device and is not proportional to the relative stiffness of the fixator and the callus. In this research, according to the cell phenotypes, the stages of the healing process were divided into six load steps in the Abaqus software. In each load step, different material properties and the same loads and boundary conditions were set. Neither bone resorption nor tissue degradation was taken into consideration.

**Figure 4:**
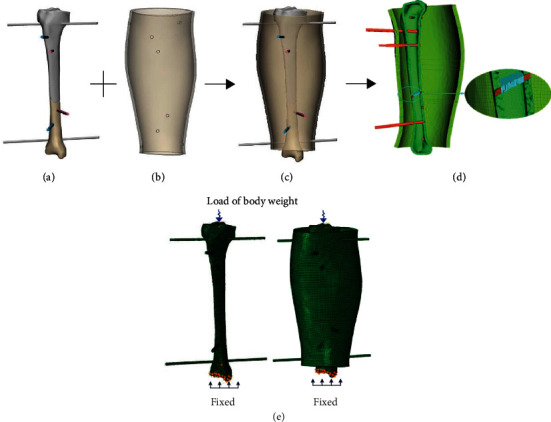
Validation of the stress distribution between external fixator and fracture gap. (a) 3D model of the fractured bone; (b) 1 cm thickness “intact shell”; (c) assembly model of the external fixator; (d) FE model of the external fixator; (e) axial load and boundary conditions applied to the FE model.

The 3D model of the pinned fracture (Figure 4(a)) after digital reduction was designed with an “intact shell” with a 1 cm thickness offset from the external contour of the shank epidermis (Figure 4(b)). Subsequently, the CAD model of the external fixator (Figure 4(c)) was imported in STP format into Hypermesh 2019.1 (Altair, Inc., France). An FE model was developed from Figure 4(d) composed of 190737 nodes and 252803 elements. The 3D model was a 2D-3D-solid map meshed by 123061 linear hexahedral elements (C3D8) and 3919 linear wedge elements (C3D6) mixed with 125823 linear tetrahedral elements (C3D4) with a characteristic length of 2 mm (Figure 4(e)). Abaqus 6.14 was used to perform the simulations of the bone healing process and tissue differentiation at the fracture gap, in order to investigate the stress distribution between the external fixator and the fracture gap during the healing process. This laid the foundation for stiffness adjustment sequence construction. The meshes of the bones and the pins shared the same nodes at their interface. It is known that the fracture gap controls the healing efficiency. Thus, a 3 mm gap (Figure 4(d)) was selected for the analysis based on the results by Son and Chang [16]. To simulate weight-bearing conditions, an axial load of 675 N was applied to the tibial plateau, while the other end was fixed in all directions (Figure 4(e)). In the Abaqus simulations, the load was set as a concentrated force on top of the tibia. Although bone is an example of a natural composite material, its properties vary from point to point. However, in this study, bone was assumed to be an isotropic material (Table 1).

### 2.3. Initial Setup of Stiffness Adjustment Tolerance

Fracture healing is a complex biological process that can be divided into primary and secondary healing [18–21]. Strain levels below 100% and above 10% are too unstable for secondary bone healing [22–24]. Strain levels below 10% permit secondary bone healing (vascularization and production of woven bone), while strain levels below 2% allow for primary bone healing. As a fracture gap shrinks with healing, the strain tolerance increases. Strain values up to 2% are tolerated by lamellar bone and up to 10% by woven bone, while above that (10% to 30%) resorption prevails [25]. It is vital to determine a stiffness adjustment tolerance for surgeons to tailor the construction of external fixator frames that suit the clinical needs of each patient and can achieve mechanical stimulation at the fracture gap. Based on existing findings, this paper assumes that during the primary bone healing stage, the stiffness of the external fixator degrades by no more than 2% at a time, while in the secondary bone healing phase, the stiffness adjustment tolerance is between 2% and 10%. This means that the strain stimulation to the callus is no more than 2% at a time during the primary bone healing stage and 2%-10% during the secondary bone healing phase, which is consistent with the reported quantitative values in existing studies. This paper summarized the stiffness adjustment tolerance and fixation rules to ensure that it meets all the previously proposed criteria (Table 2). In addition, it provides a reference range for subsequent dismantling sequence seeking and stiffness calculation.

### 2.4. Topology Optimization for Optimal Block Structure Dismantling Sequence Seeking and Corresponding Stiffness Calculation

This paper argues that the stiffness of the external fixator can be gradually decreased after the disassembly of the proposed block structure external fixator to the stiffness adjustment tolerance. Consequently, the healing callus will be stimulated by an amount of stress that corresponds to the magnitude of the stiffness adjustment tolerance. The stiffness adjustment tolerance has been determined in the previous section based on existing research, while the dismantling sequence corresponding to the tolerance of the blocks has not been defined.

**Figure 5:**
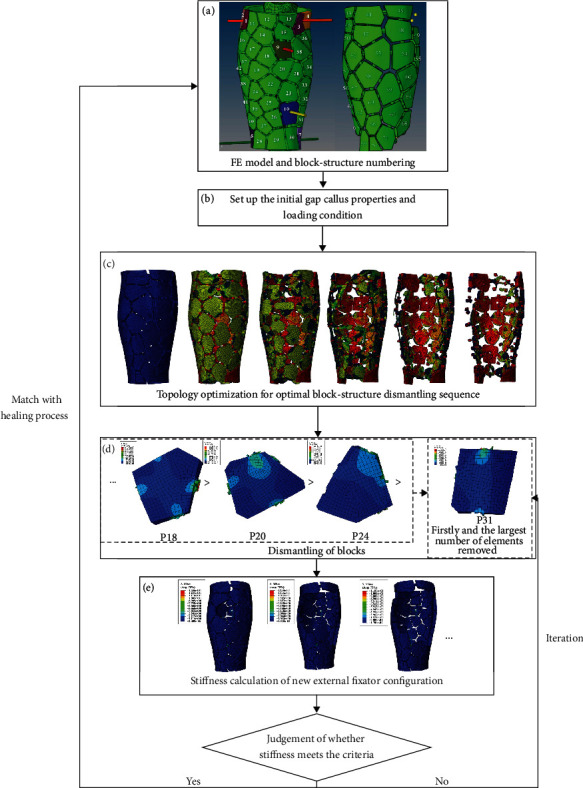
Topology optimization flow chart for optimal block structure dismantling sequence seeking and corresponding stiffness calculation. (a) FE model of the random numbered block structure external fixator; (b) properties and loading condition setup; (c) topology optimization of the entire external fixator for optimal block structure dismantling sequence; (d) dismantling of blocks; (e) stiffness calculation of new external fixator configuration;

The entire topology optimization process for optimal block structure dismantling sequence seeking and corresponding stiffness calculation is presented in Figure 5. As it can be seen in Figure 5(a), the block structure FE model was constructed based on the CAD model of the block structure external fixator (Figure 2(c)). The block structure frame was free-meshed with 2360310 eight-node tetrahedral elements (C3D8R) with a characteristic length of 2 mm. A mesh sensitivity analysis was conducted to ensure that the mesh density used in the FE model was sufficient to obtain converged numerical results. Subsequently, as it can be seen in Figure 5(b), the initial gap callus properties and loading conditions were set in accordance with Table 1. The reaction force was determined by means of weight bearing. After each modification of the material properties of the fracture gap, a new simulation was performed to simulate the fracture healing process. The values of the external fixator displacement at the point of load application were analyzed.

The actual dismantling process was simulated while ensuring convergence to meet the optimization objectives. The objective function was set to minimize the design response of the strain energy and a fraction of 20% of the initial value of the volume. The optimized area was set to the entire external fixator except for the blocks used to support the Kirschner pin (green block numbered in Figure 5(a)). Subsequently, the solid isotropic material with penalization (SIMP) method in the optimization module of Abaqus/CAE 6.14 was used to optimize the topology of the block structure. In this study, weight bearing was regarded as a concentrated force. Thus, in the topology optimization process, the minimization of the strain energy was selected as the objective function to ensure the maximum stiffness of the external fixator. Accordingly, when the stiffness remains maximum, the external fixation volume is reduced through the dismantling of the block structure external fixator. After one or more blocks have been dismantled, the stability of the fracture fixator will be reduced. Accordingly, the stiffness of the external fixator will be decreased, and the IFMs at the fracture gap will be increased to induce stress stimulation of the fracture gap. In this study, each dismantling step was performed under the premise of ensuring maximum stiffness and minimum volume. Afterwards, as it can be seen in Figure 5(d), every time a block structure external fixator was dismantled according to the result in Figure 5(c), one or more block structures were dismantled to seek the optimal dismantling sequence.

Finally, after each block structure was dismantled, the stiffness was calculated as the weight bearing axial force applied at the tibial plateau divided by the total displacement at the reference point on the top of the tibia in the tibial-fixator FE model. Subsequently, it was determined whether the stiffness meets the stiffness adjustment tolerance set previously. If yes, it matched with the healing process, the gap callus properties were set up, and the dismantling process was repeated until the block structure in the design area was completely dismantled and the result reached the goal. If not, the block structure was assembled afresh and the stiffness variation was calculated to meet the stiffness adjustment tolerance. Finally, the required dismantling sequence was obtained.

## 3. Results

### 3.1. Stress Distribution between External Fixator and Fracture Gap

**Figure 6:**
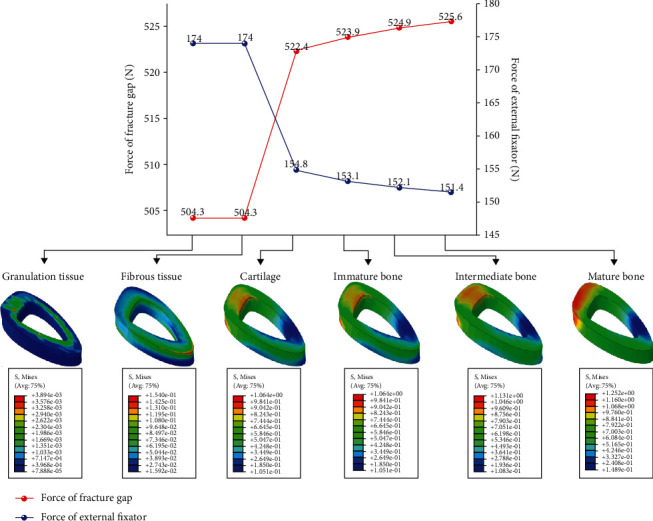
Von Mises stress distribution between fracture gap and external fixator.

An inverse relationship between the stress distribution at the fracture gap and on the external fixator was found. In the six load step (granulation tissue, fibrous tissue, cartilage, immature bone, intermediate bone, and mature bone) simulation of the fracture healing process in finite element analysis, the stress on the callus of the fracture gap increased progressively, while the stresses in the external fixator decreased continuously; The sum of the two was almost the same as that of the axial load from the weight of the patient (675 N) (Figure 6). This means that the stiffness of the healing callus is a continuously increasing parameter, which is inversely proportional to the stiffness of the fixator. Consequently, in the treatment of tibial fracture, the stiffness of the fracture callus is mutually dependent on the stiffness of the fixator. In other words, the rigidity of the selected fixation device and the amount of physiologic stresses developed by the functional activity and loading are two of the major factors that determine the mechanical milieu of a healing fracture under external fixation.

### 3.2. Stiffness Calculation and Dismantling Sequence

**Figure 7:**
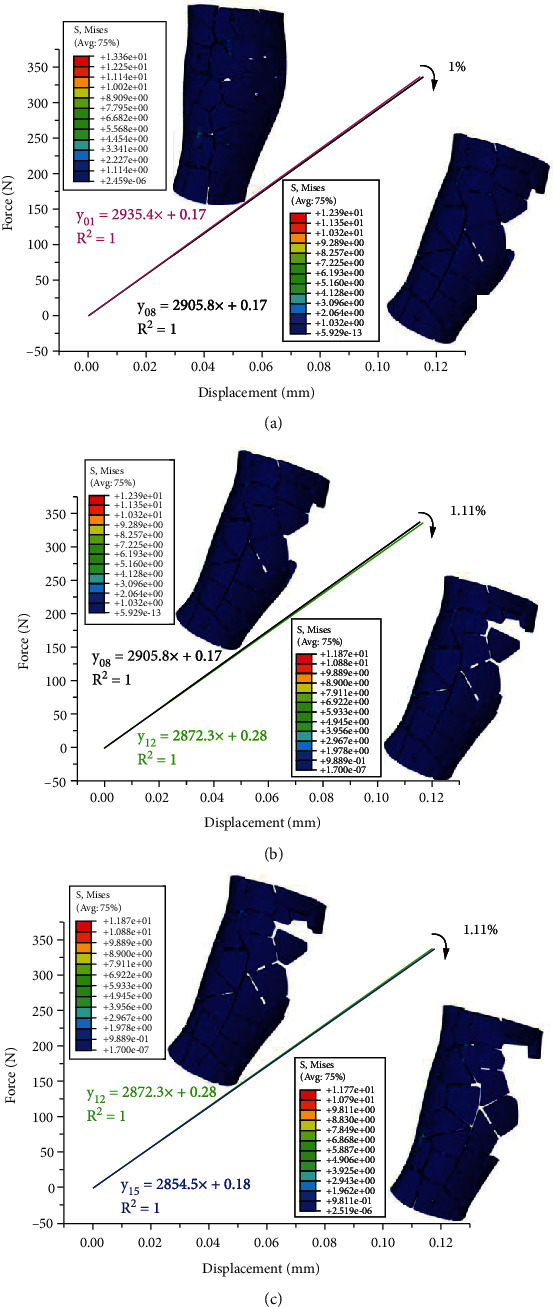
Stiffness calculation results for the optimal dismantling sequence after dismantling of block structures that are less than 2%. (a) Stiffness is reduced by 1% after dismantling of 8 block structures; (b) stiffness is reduced by 1.11% after dismantling of 4 block structures; (c) stiffness is reduced by 1.11% after dismantling of 3 block structures.

**Figure 8:**
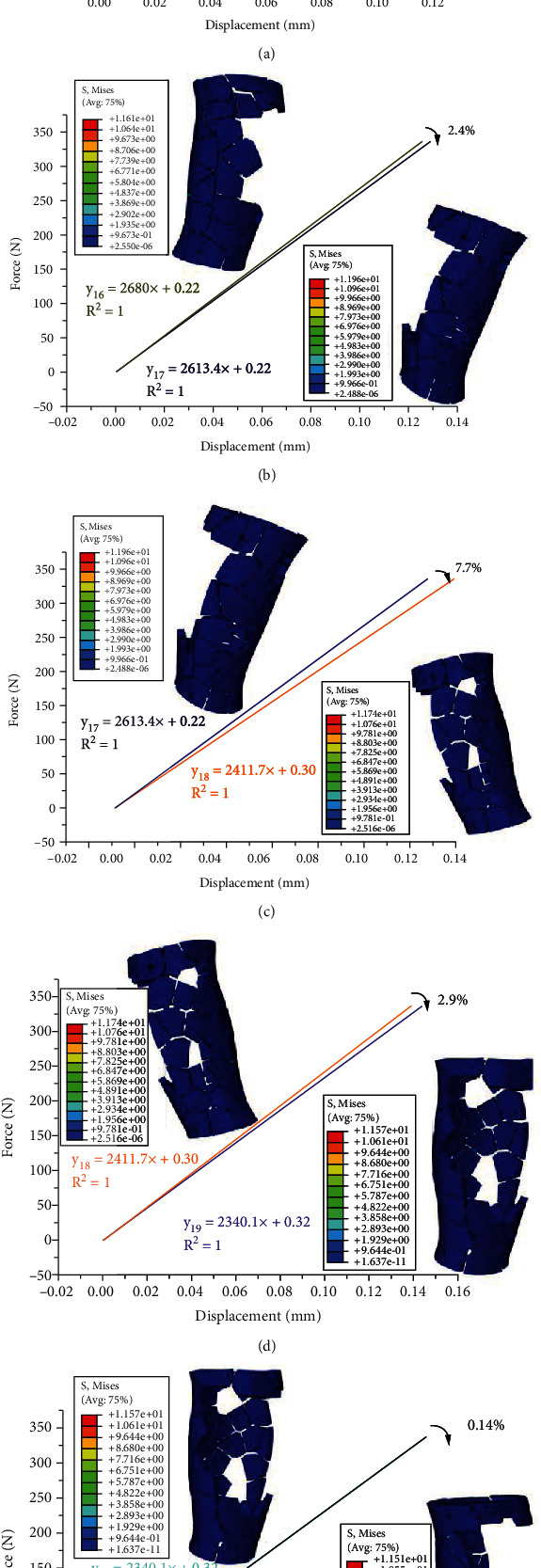
Stiffness calculation results for the optimal dismantling sequence after dismantling of block structures that are in between 2% and 10%; (a) stiffness is reduced by 6.11% after dismantling of 1 block structure; (b) stiffness is reduced by 2.4% after dismantling of 1 block structure; (c) stiffness is reduced by 7.7% after dismantling of 1 block structure; (d) stiffness is reduced by 2.9% after dismantling of 1 block structure; (e) stiffness is reduced by 0.14% after dismantling of 1 block structure.

The results indicate that a range of stiffness values can be obtained, which depends on the block structure external frame configuration and its components designed in this paper. Through optimization calculation after dismantling of block structures every time, the stiffness of external fixator can be adjusted in a sequence with 8 different levels: 1%, 1.11%, 1.11%, 6.11%, 2.4%, 7.7%, 2.9%, and 0.14% (Figures 7 and 8). According to the initially constructed sequence of stiffness adjustment tolerance presented in Table 2, there are three stiffness adjustment levels that can be used for the initial phase of healing, i.e., 1%, 1.11%, and 1.11% (Figures 7(a)–7(c)). Stiffness was degraded by 1% after dismantling of 8 blocks; after that, stiffness was degraded by 1.11% after dismantling of 4 blocks and the stiffness was degraded by 1.11% after dismantling of 3 blocks. The other four stiffness adjustment levels range from 2% to 10%, that is, 6.11%, 2.4%, 7.7%, and 2.9%, which can be used for the second phase of healing (Figures 8(a)–8(e)). The stiffness degradation of these following four steps is suitable for dismantling in the secondary bone healing stage. It should be mentioned that, in this paper, the stiffness of the external fixation was spatial stiffness.

### 3.3. Manufacture of the Adjustable Stiffness External Fixator

**Figure 9:**
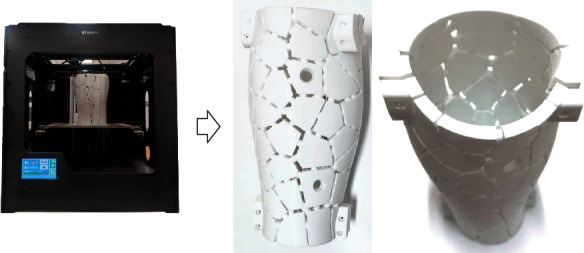
Manufacturing of the external fixator through 3D printing.

External fixator frames have been conventionally manufactured with rigid materials, such as aluminum alloys, which are typically much stiffer than the healing bone tissue. This mismatch in the elastic properties of the fixator and the healing bone tissue results in stress shielding, with the former carrying a significant proportion of the applied load and the latter lacking adequate mechanical stimuli for its remodeling. In recent years, with the progress of 3D printing and other manufacturing technologies, more and more new materials, such as polymers, composites, and metals, have been used to manufacture external fixators, facilitating the development of the whole external fixator system. In this paper, the proposed adjustable stiffness external fixator system was manufactured using fused deposition modeling (FDM), which offers a simple, reproducible, and adjustable methodology. The sliced model was inputted into the FDM 3D-printing equipment (Xi'an Huayu Electronic Technology Co. LTD, China) for physical printing where PLA (polylactic acid) was utilized as the printing material, which has a good level of both flexibility and strength. More importantly, this main material of the block structure is thermoplastic. Therefore, the column structure that connects the blocks can be dismantled using heating tools. After the physical printing was completed, the model was postprocessed, which includes removing supports, splicing parts, and polishing the surface of the model. It was observed that the printed product has a good effect and truly reflects the original concept. The FDM 3D printer and the final state of the fixator after printing are shown in Figure 9.  Table 3 lists the manufacturing parameters in detail.

## 4. Discussion

Whether it is safe to remove the external fixators or not requires an objective assessment of bone healing while the fixator is still in place. In many circumstances, although radiographic assessment of fracture callus formation has grown into shape, doctors still are not certain enough of completely removing the external fixation. This is due to fear that sudden complete removal of the external fixation would cause secondary damage. Therefore, patients are required to wear it for an additional period of time. The external fixator configuration proposed in this paper can well tackle this problem. With the growth of the callus, a well-matched dismantling of the block structure external fixator is performed, and the goal is that the stiffness of the fixator is always compatible with that of the callus until complete fracture healing. A not entirely dismantled block structure can not only adapt to the stiffness of the growing callus but also play a certain protective role. Controlled IFM can be imposed very early after the placement of the fixator frame, when mechanical stimulation is most effective, while the active loading by the patient is least [31]. Therefore, it can be possible to set the start time of stiffness adjustment earlier.

The fixator proposed in this research is a dynamic modulating stiffness construct that can be adjusted throughout the repair process. This design enables the medical staff to regulate the mechanical environment (stiffness) of the fracture gap in the clinical treatment of tibial fracture. Some published papers reported that the stiffness of the external fixator can be adjusted while the device is attached to the patient [32–37]. However, most of the designs lack the function of valid dynamic stiffness adjustment based on parameterized stiffness sequence, and their clinical applications are limited by high cost and complexity in operation. Compared to the design of the existing fixators in the market, the concept presented in this paper has accurate quantitative adjustment capacity, which can dismantle the block structures of the fixator that are connected to each other by bars according to a preestablished dismantling sequence based on the stiffness adjustment tolerance of each patient, and the mechanical stimulus can be adapted at different stages of the healing process. Like the findings of other computational modeling research for fracture healing [17, 26, 38, 39], the finite element analysis via the six load step simulation of the fracture healing process validated an inverse relationship between the stress distribution at the fracture gap and on the external fixator. Furthermore, as shown in Figures 7 and 8, fracture movement of this concept can be regulated in a sequence with 8 different levels according to the healing phase. The design method of the adjustable external fixator can be used in various studies of stiffness regulation related to fracture healing and can promote the discovery of new therapies not only for complex fractures but also for the treatment of standard fractures, since conformable mechanical stimulus can accelerate the healing process. This methodology also provides a good basis for the further development of variable stiffness design. The advantages of accurate reduction, minimal invasion, stable fixation, and timely stress adjustment can improve the healing of long bone shaft fractures. In addition, the proposed device is easy to handle, which can shorten the operation time, prevent repeated exposure to radiation, and require less experience from the surgeons. Consequently, it has a bright prospect for clinical application.

PLA (polylactic acid) was utilized as the printing material, which is low-cost and has a good level of both flexibility and strength. However, time required to simulate and print via FDM additive manufacturing technology may limit the clinical applications of this proposed concept. However, with the development of the printing process and computation tools, the time required to design and manufacture would be significantly improved.

There were some limitations in this study. Simulation of the fracture healing process and stiffness calculation for the optimal dismantling sequence after dismantling of block structures are purely computational representations of a biological healing model. Despite all that, reduction and fixation are clinically applied, but clinical validation of stiffness degradation needs to be done later. Future research will be concentrated both on improving of the FE model and clinical aspects of the concept. In parallel, many efforts will be done to completely develop the totally automated design and optimization process and relative computer-aided design software for the customized adjustable stiffness external fixator. In the future application, only the CT scanning data and the body weight are required to generate the FEA simulation model and the adjustable stiffness external fixator configuration, based on which the final customized block structure fixator for printing can be obtained through an automated optimization procedure.

## 5. Conclusions

This paper presented a novel patient-specific external fixator with an adjustable stiffness function developed through customized design and fabrication. To the best of our knowledge, this is the first reported patient-specific adjustable stiffness external fixator for mechanically stimulated healing of tibial fractures.

1. A method for designing patient-specific adjustable stiffness external fixator was proposed
2. The FE simulations revealed that there is an inverse relationship between the stress distribution at the fracture gap and on the external fixator. Therefore, for the external fixation used in the treatment of tibial fractures, the stiffness of the fracture callus is mutually dependent on that of the fixator
3. Based on the pathological characteristics of the patient in this research, 8 levels of stiffness were applied for mechanical stimulus adaption. The dismantling sequence was provided through a numbered block structure external fixator

## Figures and Tables

**Table 1:** Material properties of tissues and fixator components [17].

Tissue type	Young's modulus (MPa)	Poisson's ratio
Granulation/connective tissue	0.2	0.167
Fibrous tissue	2	0.167
Cartilage	10	0.167
Immature/woven bone	1000	0.3
Mature bone	6000	0.3
Cortical bone	20000	0.3
Wires	210000	0.3
Fixator	8300	0.28

**Table 2:** Criteria for strain and fixation stability of the callus at different healing stages.

Classification of various healing processes [16, 17, 26]	Granulation tissue	Fibrous tissue	Cartilage	Immature bone	Intermediate bone	Mature bone	Cortical bone
	Primary healing Direct bone repair without endochondral ossification			Secondary healing Appropriate stability of the fracture site to maintain the biological healing response			
	Inflammatory stage	Anabolic phase		Endochondral stage			
				Catabolic phase			
					Coupled remodeling		
	Inflammatory response [5, 27]	Soft callus formation		Hard callus development	Remodeling		
Fix condition [5, 18, 24, 25, 27, 28]	Absolute stability		Relative stability				
Young's modulus, *E* (MPa) [17, 29]	*E* ≤ 0.02	*E* ≤ 5	*E* ≤ 500	*E* ≤ 1000	*E* ≤ 2000	*E* ≤ 6000	*E* ≤ 8000
Day [30]	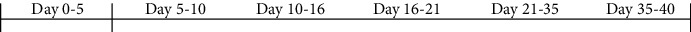						
Stiffness adjustment tolerance of the fixator	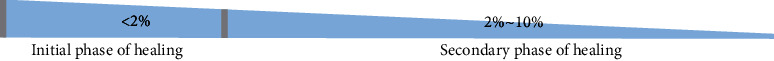						

**Table 3:** Printing parameters of FDM for the customized external fixator.

Printing parameters	Value
Nozzle size (mm)	0.4
Printing speed (mm/s)	30
Layer height (mm)	0.2
Shell thickness (mm)	0.4
Printing temperature (°C)	220
Fill density (%)	100

## Data Availability

The CAD and finite element data used to support the findings of this study were supplied by Honghui Hospital, Xi'an Jiaotong University, under license and so cannot be made freely available. Request for access to these data should be made to Fen Qiao, qiaofen7502@163.com.
